# Trending prevalence of healthcare-associated infections in a tertiary hospital in China during the COVID-19 pandemic

**DOI:** 10.1186/s12879-022-07952-9

**Published:** 2023-01-20

**Authors:** Rong Rong, Lanxi Lin, Yongjie Yang, Shumin Zhao, Ruiling Guo, Junpeng Ye, Xinghua Zhu, Qiong Wen, Dayue Liu

**Affiliations:** 1 Department of Nosocomial Infection, The First Affiliated Hospital, Sun Yat-sen University, Zhong Shan 2nd Road, No. 58, Guangzhou, 510080 Guangdong China; 2 Department of Nephrology, The First Affiliated Hospital, Sun Yat-sen University, Guangzhou, China; 3 Key Laboratory of Guangdong Province, Key Laboratory of National Health Commission, Zhong Shan 2nd Road, No. 58, Guangzhou, 510080 Guangdong China

**Keywords:** Healthcare-associated infection, Prevalence, COVID-19, Seasonal variation

## Abstract

**Background:**

The purpose of this study was to demonstrate both the four-year prevalence trend of healthcare-associated infections (HAIs) in a large tertiary hospital and the trend regarding the prevalence of HAIs following the outbreak of coronavirus disease 2019 (COVID-19) in order to provide evidence of hospital infection management during the COVID-19 pandemic.

**Methods:**

Based on the hospital’s electronic nosocomial infection databases related to HAIs, we retrospectively identified the HAI cases to assess the epidemiological characteristics of HAIs from January 1, 2018, to December 31, 2021, in a large tertiary hospital in China. Similarly, the trends of HAIs after the COVID-19 outbreak and the seasonal variation of HAIs were further analyzed.

**Results:**

The HAI cases (*n* = 7833) were identified from the inpatients (*n* = 483,258) during the 4 years. The most frequently occurring underlying cause of HAIs was respiratory tract infections (44.47%), followed by bloodstream infections (11.59%), and urinary tract infections (8.69%). The annual prevalence of HAIs decreased from 2.39% in 2018 to 1.41% in 2021 (*P* = 0.032), with the overall prevalence of HAIs significantly decreasing since the outbreak of COVID-19 (2.20% in 2018–2019 vs. 1.44% in 2020–2021, *P* < 0.001). The prevalence of respiratory tract infections decreased most significantly; whereas, overall, the prevalence of HAIs was significantly greater during the winter compared with the rest of the year.

**Conclusions:**

Not only did the annual prevalence of HAIs decrease from 2018 to 2021, but it also significantly decreased since the start of the COVID-19 pandemic, particularly respiratory tract infections. These results provide evidence for the need to prevent HAIs, especially during the winter season.

**Supplementary Information:**

The online version contains supplementary material available at 10.1186/s12879-022-07952-9.

## Introduction

Healthcare-associated infection (HAI) is a global public health issue, which leads to a prolonged hospital stay, increased antimicrobial resistance, additional healthcare expenditures, as well as a high mortality [1, 2]. Previous surveys have revealed that the annual financial burden of HAIs was about $6.5 billion in the USA and up to €7.0 billion in Europe between 1995 and 2010, and HAIs also cause high financial losses in developing countries every year [1, 3]. A systematic review of studies conducted in general hospitals in China has suggested that the pooled median estimates of the total medical expenditures and hospitalization days per inpatient were nearly $4000 (USD) (¥24,881.37) more and 13.89 days longer in patients with HAI than in patients without HAI [4]. Thus, it is particularly important for hospital administrators to pay more attention to the prevention of nosocomial infection.

In China, the weighted prevalence of HAI in 2018 was 3.13% in tertiary and specialized hospitals [2]. Importantly, the overall prevalence of HAI in surveyed Chinese hospitals was less than those of previous reports from the USA (4.0%) [5], the European Union and the European Economic Area (5.9%) [6], and Southeast Asia (9.0%) [7].

Interestingly, the overall prevalence of HAI was also different depending on the region of China. For instance, in Guangdong Province, 1.24% of inpatients had at least one HAI between June 2017 and May 2018 [8], whereas the overall prevalence of HAI was 2.10% in 2014 in Beijing City [9], 2.41% in 2014 in Guizhou Province [10], and 3.88% in 2007–2008 in Hubei Province [11]. However, it is not completely clear whether the differences in the prevalence of HAI in different regions of China are influenced by climate conditions or whether the prevalence of HAI varies according to different outdoor temperatures.

Among the 52 Chinese hospitals surveyed, the most frequently occurring causes of HAI were lower respiratory tract infections (47.2%), urinary tract infections (12.3%), upper respiratory tract infections (11.0%), and surgical site infections (6.2%) [12]. A previous study also has revealed that the addition of bioaerosol treatment and coronavirus disease 2019 (COVID-19) mitigation measures significantly reduced airborne ultrafine particles and altered the bioburden of hospital environments since the outbreak of COVID-19 [13]. However, that study was conducted with a number of uncontrollable variables and lacked a concurrent control group. It is unclear whether the prevalence of HAI in Chinese medical institutions has been affected by COVID-19. Therefore, this study aimed to demonstrate the prevalence trend of all types of HAI while exploring the seasonal variation of its prevalence over a four-year study period at The First Affiliated Hospital of Sun Yat-sen University, Guangzhou, China. Additionally, the difference in the overall prevalence of HAI between before and after the COVID-19 outbreak is also described herein.

## Material and methods

### Study design and setting

In this study, a retrospective observational study was performed from January 1, 2018, to December 31, 2021, in a large tertiary hospital with a total of 3523 beds in Guangzhou, China. Real-time surveillance of HAIs with an online nosocomial infection surveillance system was carried out to monitor all patients during their hospital stay. Ethical approval was obtained from The First Affiliated Hospital of Sun Yat-sen University ([2022]262).

This study included patients who had been hospitalized for more than 48 h between January 1, 2018, and December 31, 2021. The patients from outpatient services or day surgery centers were excluded from this study.

According to the Nosocomial Infection Diagnostic Criteria published in 2001 by the National Health Commission of the People’s Republic of China [14], HAI is defined as an infection that occurs 48 h after a patient had been admitted to the hospital. In this study, the following conditions were also considered as HAIs: (1) the patient was admitted with an infection associated with a previous hospitalization, and the time interval between the previous discharge and this readmission was less than 24 h; (2) neonate-acquired infections during delivery; (3) a medical invasive device was inserted on day 1 or day 2 of admission, which resulted in any element of the infection criteria present within 48 h.

In this study, the types of HAIs included respiratory tract infections, ventilator-associated pneumonia, pleural cavity infections, bloodstream infections, urinary tract infections, catheter-associated urinary tract infections, surgical site infections, intra-abdominal infections, gastrointestinal infections, organ or lacuna infections, deep surgical site infections, intracranial infections, skin and soft-tissue infections, oral cavity infections, and cardiovascular system infections, among others.

### Data collection

The inpatient infection-related information was collected using an automatic online real-time nosocomial infection surveillance system, which automatically screens for potential HAIs. Moreover, the fever history, microbiological reports, serological and molecular testing results, radiological information, and antibiotic usage were inputted into the system by algorithms to screen for potential HAI.

The clinical information collected for each inpatient included the following: demographic characteristics, hospitalization days, diagnosis, antibiotic treatment, surgery data, Intensive Care Unit (ICU) admission, specific device days, and body temperature; this information was obtained from the hospital information system. In addition, the microbiologic profile and routine test results were obtained from the laboratory information system. Moreover, the radiology reports were obtained from the radiology information system.

All reported cases of HAI that occurred during hospitalization were identified by infectious disease specialists and doctors to ensure the accurate identification of the HAI cases. Meanwhile, to ensure that the collected data were valid and reliable, the infectious disease specialist’s team checked the collected data and removed invalid inputs.

### Data analysis

The prevalence of HAI was calculated as the number of new HAIs per 100 patient-days (HAI prevalence = Number of HAIs/100 patient-days). Relative proportions were defined as each infection site versus all HAIs per year. The incidence rate ratios (IRRs) of seasonal variation were calculated separately for the winter (December 1 to February 28) and the nine remaining months during 2018–2021.

SAS 9.4 and R version 4.1.1 were used for all data processing and data analysis. Continuous variables were compared using the rank-sum test. Categorical variables were compared by using the chi-squared test. The standardized mean difference (SMD) of variables between two groups was reported to evaluate the balance of covariate distribution between different population groups [15, 16]. SMD < 0.1 was considered appropriate balance of variables between two groups [16]. Continuous variables with a normal distribution were expressed as the mean ± standard deviation, and those without a normal distribution were expressed as the median and interquartile range. Categorical variables were described as the frequency and percentage, and linear regression models were used to evaluate the trend association of HAI during the year. Furthermore, cross-correlation analysis was performed using the ccf function in R to assess the relationship between monthly meteorological data and the monthly prevalence of HAI. Statistically significant differences were defined by a *P*-value of < 0.05.

## Results

### Patient population

The demographic and clinical characteristics of all discharged patients from January 1, 2018, to December 31, 2021, are shown in Table 1 and Additional file 1: Table S1. During the four-year study period, the inpatient admissions (*n* = 483,258) were eligible for inclusion in this study, including the inpatients with HAI (*n* = 7833) and the inpatients without HAI (*n* = 475,425). Males accounted for the larger proportion (59.2%) in the HAI group, and the patients in the HAI group were slightly older (49.0 years vs. 48.0 years, *P* < 0.001). Compared to the non-HAI group, the length of hospital stay was much longer in the HAI group (22.0 days vs. 5.0 days, *P* < 0.001), whereas the proportion of ICU patients in the HAI group was much greater than that of the non-HAI group (29.1% vs. 4.3%, *P* < 0.001). Among the patients in the HAI group, 27.2% received surgery and 90.7% received antibiotics (Table 1).

**Table 1:** Demographic and clinical characteristics of all discharged patients 2018–2021

Variables	Total n = 483,258	Without HAI n = 475,425	With HAI n = 7833	*P*-value
Age, median (IQR)	48.0 (32.0,61.0)	48.0 (32.0,61.0)	49.0 (22.0,64.0)	< 0.001
Gender				< 0.001
Female	252,479 (52.2%)	249,282 (52.4%)	3197 (40.8%)	
Male	230,779 (47.8%)	226,143 (47.6%)	4636 (59.2%)	
Length of stay, median (IQR)	5.0 (2.0,9.0)	5.0 (2.0,9.0)	22.0 (13.0,33.0)	< 0.001
ICU				< 0.001
No	460,642 (95.3%)	455,086 (95.7%)	5556 (70.9%)	
Yes	22,616 (4.7%)	20,339 (4.3%)	2277 (29.1%)	
Receiving surgery				< 0.001
No	381,435 (78.9%)	375,735 (79.0%)	5700 (72.8%)	
Yes	101,823 (21.1%)	99,690 (21.0%)	2133 (27.2%)	
Receiving antibiotics				< 0.001
No	301,603 (62.4%)	300,874 (63.3%)	729 (9.3%)	
Yes	181,655 (37.6%)	174,551 (36.7%)	7104 (90.7%)	

*IQR*  interquartile range. Continuous variables were compared using the rank-sum test. Categorical variables were compared by using the Chi-square test

### Prevalence and distribution of HAI

**Fig. 1:**
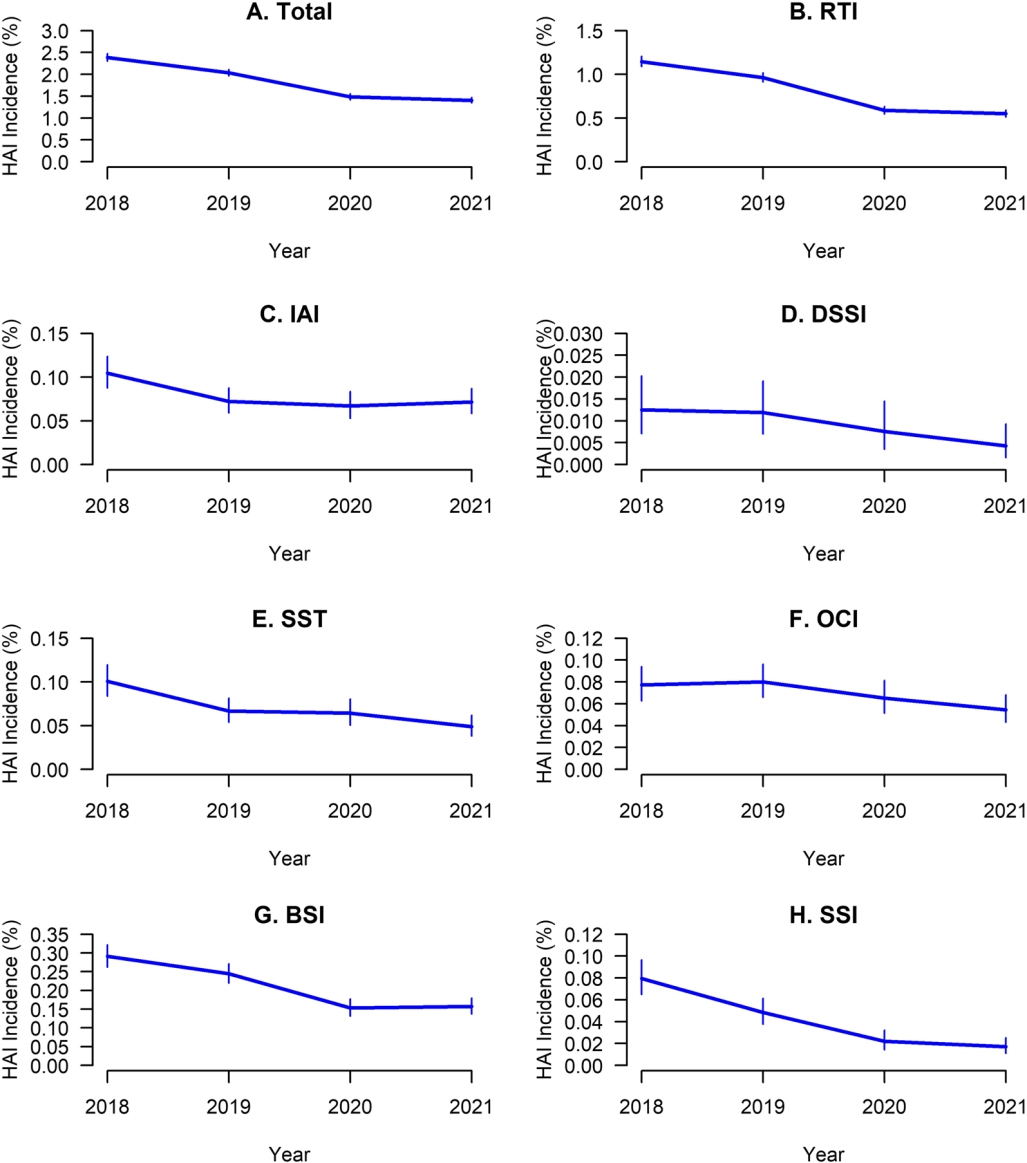
Yearly HAI prevalence, 2018–2021. **A** Total HAIs; **B**–**H** Seven types of HAIs were significantly decreased after the start of the COVID-19 pandemic. *P* < 0.05. *HAI* healthcare-associated infection, *RTI* respiratory tract infection, *IAI* intra-abdominal infection, *DSSI* deep surgical site infection, *SST* skin and soft-tissue infection, *OCI* oral cavity infection, *BSI* bloodstream infection, *SSI* surgical site infection

Bearing in mind that a total of 7833 HAI patients were identified between 2018 and 2021, the annual prevalence of HAI significantly decreased from 2.39% in 2018 to 1.41% in 2021 (Trend *P* = 0.032) and was the most pronounced between 2019 and 2020 (Fig. 1 and Table 2).

**Table 2:** Prevalence of different HAI types (rate), 2018–2021

	2018	2019	2020	2021	Trend test		2018–2019 vs 2020–2021
					Coefficient (CI 95%)	*P*-value	*P*-value
Total	2.39 (2.3, 2.47)	2.04 (1.97, 2.11)	1.49 (1.42, 1.56)	1.41 (1.35, 1.47)	− 0.35 (− 0.63 to − 0.07)	0.032	< 0.001
RTI	1.15 (1.09, 1.21)	0.96 (0.91, 1.02)	0.59 (0.55, 0.63)	0.55 (0.51, 0.59)	− 0.22 (− 0.41 to − 0.03)	0.039	< 0.001
VAP	0.03 (0.02, 0.04)	0.02 (0.01, 0.02)	0.02 (0.01, 0.02)	0.01 (0.01, 0.02)	0 (− 0.01 to 0)	0.153	0.064
PCI	0.02 (0.01, 0.03)	0.01 (0.01, 0.02)	0.01 (0, 0.01)	0.01 (0.01, 0.02)	0 ( − 0.01 to 0.01)	0.646	0.185
UTI	0.17 (0.14, 0.19)	0.17 (0.14, 0.19)	0.16 (0.14, 0.18)	0.15 (0.13, 0.17)	− 0.01 (− 0.01 to 0)	0.055	0.233
CAUTI	0.01 (0.01, 0.02)	0.01 (0, 0.01)	0.01 (0, 0.01)	0.01 (0.01, 0.02)	0 (− 0.01 to 0.01)	0.666	0.792
IAI	0.1 (0.09, 0.12)	0.07 (0.06, 0.09)	0.07 (0.05, 0.08)	0.07 (0.06, 0.09)	− 0.01 (− 0.04 to 0.02)	0.223	0.019
GI	0.12 (0.1, 0.14)	0.12 (0.1, 0.14)	0.11 (0.09, 0.13)	0.09 (0.08, 0.11)	− 0.01 (− 0.02 to 0)	0.096	0.051
OLI	0.01 (0, 0.01)	0 (0, 0.01)	0 (0, 0)	0 (0, 0.01)	0 ( − 0.01 to 0)	0.667	0.524
DSSI	0.01 (0.01, 0.02)	0.01 (0.01, 0.02)	0.01 (0, 0.01)	0 (0, 0.01)	0 (− 0.01 to 0)	0.035	0.014
ICI	0.01 (0.01, 0.02)	0.01 (0, 0.01)	0.01 (0, 0.02)	0 (0, 0.01)	0 (− 0.01 to 0)	0.088	0.073
SST	0.1 (0.08, 0.12)	0.07 (0.05, 0.08)	0.06 (0.05, 0.08)	0.05 (0.04, 0.06)	− 0.02 (− 0.03 to 0)	0.067	< 0.001
OCI	0.08 (0.06, 0.09)	0.08 (0.07, 0.1)	0.07 (0.05, 0.08)	0.05 (0.04, 0.07)	− 0.01 (− 0.02 to 0)	0.088	0.008
CVS	0.01 (0.01, 0.02)	0.01 (0, 0.01)	0.01 (0, 0.01)	0.01 (0, 0.01)	0 (− 0.01 to 0)	0.25	0.114
BSI	0.29 (0.26, 0.32)	0.24 (0.22, 0.27)	0.15 (0.13, 0.18)	0.16 (0.14, 0.18)	− 0.05 (− 0.1 to 0.01)	0.061	< 0.001
SSI	0.08 (0.06, 0.1)	0.05 (0.04, 0.06)	0.02 (0.01, 0.03)	0.02 (0.01, 0.03)	− 0.02 (− 0.04 to 0)	0.038	< 0.001
Others	0.2 (0.17, 0.22)	0.21 (0.19, 0.24)	0.21 (0.18, 0.23)	0.21 (0.18, 0.23)	0 ( − 0.01 to 0.02)	0.756	0.924

Trend test: 2018 to 2021 HAI change trend, linear regression analysis; 2018–2019 vs 2020–2021 means before and after COVID-19 pandemic. *BSI* bloodstream infection, *CAUTI* catheter-associated urinary tract infection, *CVS* cardiovascular system infection, *GI* gastrointestinal infection, *IAI* intra-abdominal infection, *ICI* intracranial infection, *OCI* oral cavity infection, *OLI* organ lacuna infection, *DSSI* deep surgical site infection, *PCI* pleural cavity infection, *RTI* respiratory tract infection, *SSI* surgical site infection, *SST* skin and soft-tissue infection, *UTI* urinary tract infection, *VAP* ventilator-associated pneumonia

**Fig. 2:**
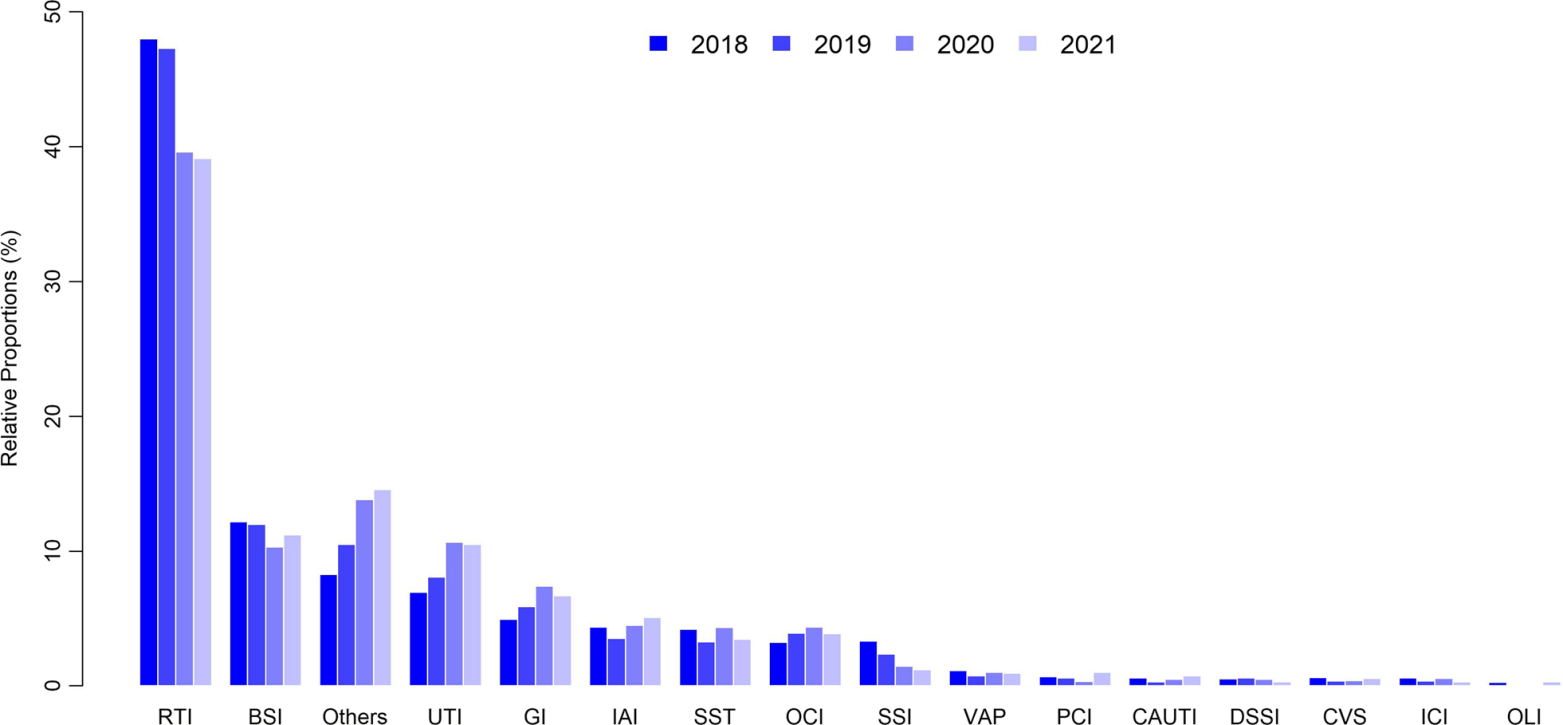
Distribution of infection types. The four columns of each infection type represent the proportions of infections in 2018–2021, respectively. The top five infection types in each year were the same, but there were changes in the proportions between before and after the COVID-19 pandemic

During the four-year study period, the most frequently occurring types of HAI were respiratory tract infections (*n* = 4312, 44.47%), followed by bloodstream infections (*n* = 1124, 11.59%), urinary tract infections (*n* = 843, 8.69%), and gastrointestinal infections (*n* = 586, 6.04%) (Fig. 2 and Table 3). Meanwhile, the results revealed a significantly declining trend in respiratory tract infections (Trend *P* = 0.039), surgical site infections (Trend *P* = 0.038), and deep surgical site infections (Trend *P* = 0.035). However, no significant changes in the prevalence of other types of HAI were observed (Table 2).

**Table 3:** The relative proportion by infection site (%), 2018–2021

	2018	2019	2020	2021	Total
Total	3060 (100)	2902 (100)	1754 (100)	1981 (100)	9697 (100)
RTI	1469 (48.01)	1373 (47.31)	695 (39.62)	775 (39.12)	4312 (44.47)
VAP	35 (1.14)	22 (0.76)	18 (1.03)	19 (0.96)	94 (0.97)
PCI	21 (0.69)	17 (0.59)	6 (0.34)	20 (1.01)	64 (0.66)
UTI	213 (6.96)	235 (8.1)	187 (10.66)	208 (10.5)	843 (8.69)
CAUTI	18 (0.59)	9 (0.31)	9 (0.51)	15 (0.76)	51 (0.53)
IAI	134 (4.38)	103 (3.55)	79 (4.5)	101 (5.1)	417 (4.3)
GI	152 (4.97)	171 (5.89)	130 (7.41)	133 (6.71)	586 (6.04)
OLI	8 (0.26)	2 (0.07)	1 (0.06)	6 (0.3)	17 (0.18)
DSSI	16 (0.52)	17 (0.59)	9 (0.51)	6 (0.3)	48 (0.49)
ICI	18 (0.59)	11 (0.38)	10 (0.57)	6 (0.3)	45 (0.46)
SST	129 (4.22)	95 (3.27)	76 (4.33)	69 (3.48)	369 (3.81)
OCI	99 (3.24)	114 (3.93)	77 (4.39)	77 (3.89)	367 (3.78)
CVS	19 (0.62)	11 (0.38)	7 (0.4)	11 (0.56)	48 (0.49)
BSI	373 (12.19)	348 (11.99)	181 (10.32)	222 (11.21)	1124 (11.59)
SSI	102 (3.33)	69 (2.38)	26 (1.48)	24 (1.21)	221 (2.28)
Others	254 (8.3)	305 (10.51)	243 (13.85)	289 (14.59)	1091 (11.25)

*BSI* bloodstream infection, *CAUTI* catheter-associated urinary tract infection, *CVS* cardiovascular system infection, *GI* gastrointestinal infection, *IAI* intra-abdominal infection, *ICI* intracranial infection, *OCI* oral cavity infection, *OLI* organ lacuna infection, *DSSI* deep surgical site infection, *PCI* pleural cavity infection, *RTI* respiratory tract infection, *SSI* surgical site infection, *SST* skin and soft-tissue infection, *UTI* urinary tract infection, *VAP* ventilator-associated pneumonia

Since the outbreak of COVID-19 at the end of 2019, the overall prevalence of HAI decreased significantly (2.20% in 2018–2019 vs. 1.44% in 2020–2021, *P* < 0.001), with the prevalence of respiratory tract infections (1.05% in 2018–2019 vs. 0.57% in 2020–2021, *P* < 0.001), bloodstream infections (0.27% in 2018–2019 vs. 0.16% in 2020–2021, *P* < 0.001), and surgical site infections decreasing the most significantly (0.06% in 2018–2019 vs. 0.02% in 2020–2021, *P* < 0.001) (Table 2).

### Seasonal variation

**Fig. 3:**
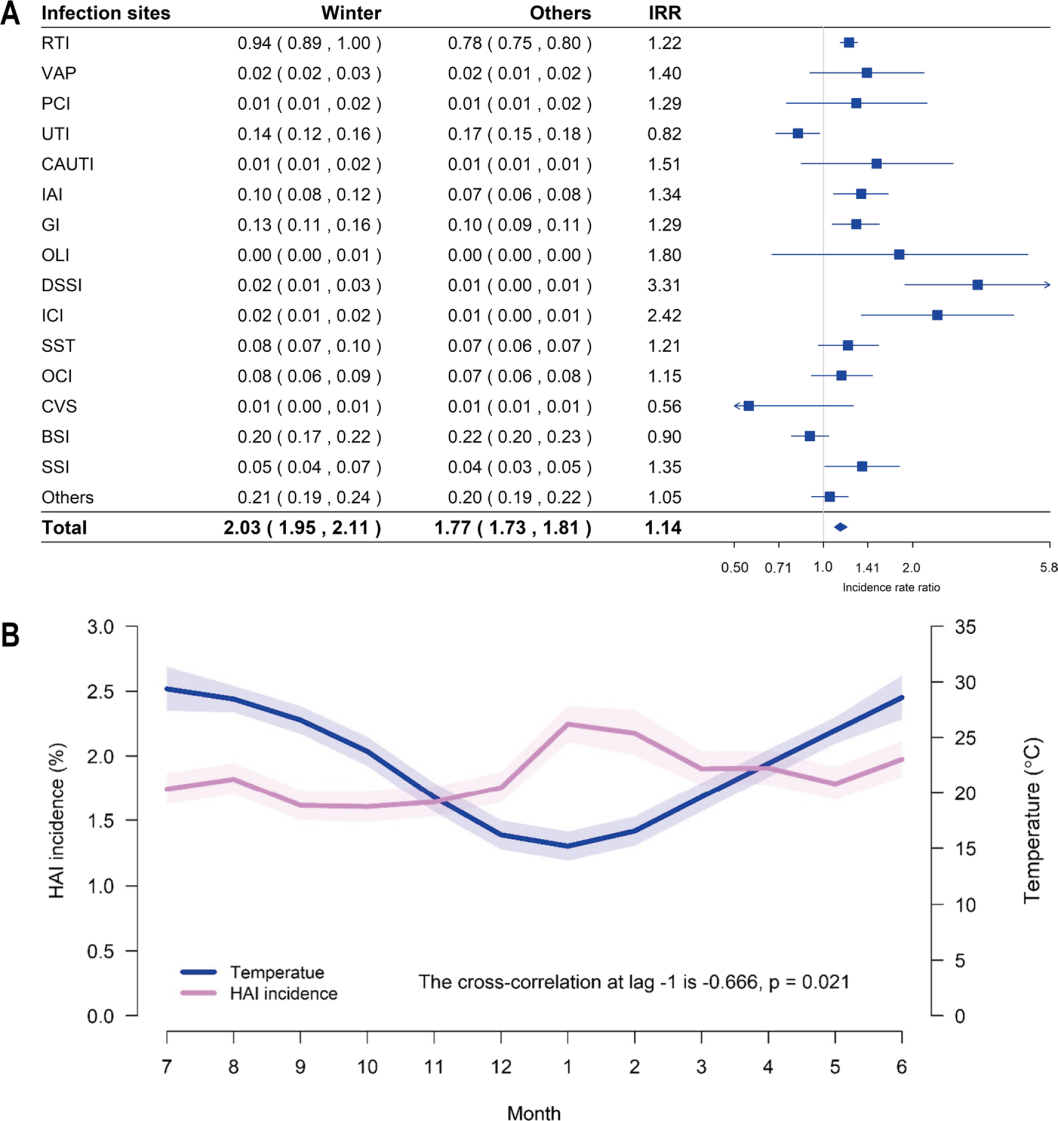
**A** Forest plot of the prevalence rates of healthcare-associated infection (HAI) during the winter (December–February) compared with the rest of the year, 2018–2021. IRR, incidence rate ratio. *CI* confidence interval. **B** Monthly HAI prevalence and temperature–time series plot. It can be seen that the infection rate increases with the decrease of temperature, while the infection rate decreases with the increase of temperature. The infection rate forms a small peak in the winter. The light color on the figure indicates the 95% confidence interval

By comparing the prevalence of HAI between the winter months (from December to February) and the rest of the year, we found that the overall prevalence was greater in the winter (IRR: 1.14, 95% confidence interval (CI): 1.09–1.20) (Table 4). Similarly, the respiratory tract infections (IRR: 1.22, 95% CI: 1.14–1.30), gastrointestinal infections (IRR: 1.29, 95% CI: 1.07–1.59), intra-abdominal infections (IRR: 1.34, 95% CI: 1.08–1.65), surgical site infections (IRR: 1.35, 95% CI: 1.01–1.80), deep surgical site infections (IRR: 3.31, 95% CI: 1.88–5.82), and intracranial infections (IRR: 2.42, 95% CI: 1.34–4.37) had a higher prevalence; whereas urinary tract infections (IRR: 0.82, 95% CI: 0.69–0.97) had a lower prevalence during the same season (Fig. 3A and Table 4).

**Table 4:** Incidence rates of HAI during winter (Dec–Feb) compared with the rest of the year, 2018–2021

	Winter	Others	IRR (95 CI%) Winter vs. others
Total	2.03 (1.95, 2.11)	1.77 (1.73, 181)	**1.14 (1.09–1.2)**
RTI	0.94 (0.89, 1)	0.78 (0.75, 0.8)	**1.22 (1.14–1.3)**
VAP	0.02 (0.02, 0.03)	0.02 (0.01, 0.02)	1.4 (0.9–2.18)
PCI	0.01 (0.01, 0.02)	0.01 (0.01, 0.02)	1.29 (0.75–2.23)
UTI	0.14 (0.12, 0.16)	0.17 (0.15, 0.18)	**0.82 (0.69–0.97)**
CAUTI	0.01 (0.01, 0.02)	0.01 (0.01, 0.01)	1.51 (0.84–2.73)
IAI	0.1 (0.08, 0.12)	0.07 (0.06, 0.08)	**1.34 (1.08–1.65)**
GI	0.13 (0.11, 0.16)	0.1 (0.09, 0.11)	**1.29 (1.07–1.54)**
OLI	0 (0, 0.01)	0 (0, 0)	1.8 (0.67–4.88)
DSSI	0.02 (0.01, 0.03)	0.01 (0, 0.01)	**3.31 (1.88–5.82)**
ICI	0.02 (0.01, 0.02)	0.01 (0, 0.01)	**2.42 (1.34–4.37)**
SST	0.08 (0.07, 0.1)	0.07 (0.06, 0.07)	1.21 (0.96–1.53)
OCI	0.08 (0.06, 0.09)	0.07 (0.06, 0.08)	1.15 (0.91–1.46)
CVS	0.01 (0, 0.01)	0.01 (0.01, 0.01)	0.56 (0.25–1.26)
BSI	0.2 (0.17, 0.22)	0.22 (0.2, 0.23)	0.9 (0.78–1.04)
SSI	0.05 (0.04, 0.07)	0.04 (0.03, 0.05)	**1.35 (1.01–1.8)**
Others	0.21 (0.19, 0.24)	0.2 (0.19, 0.22)	1.05 (0.91–1.21)

The bold values mean *P* < 0.05. *BSI* bloodstream infection, *CAUTI* catheter-associated urinary tract infection, *CVS* cardiovascular system infection, *GI* gastrointestinal infection, *IAI* intra-abdominal infection, *ICI* intracranial infection, *OCI* oral cavity infection, *OLI* organ lacuna infection, *DSSI* deep surgical site infection, *PCI* pleural cavity infection, *RTI* respiratory tract infection, *SSI* surgical site infection, *SST* skin and soft-tissue infection, *UTI* urinary tract infection, *VAP* ventilator-associated pneumonia

Moreover, our meteorological data showed that the three winter months, i.e., from December to February, were the months with the lowest outdoor temperature in Guangzhou, China. The cross-correlation coefficients were compared to explore the relationship between the monthly temperature data and the monthly prevalence of HAI, and the results revealed that HAI prevalence was negatively correlated with the outdoor temperature (cross-correlation at lag 0: − 0.492, *P* = 0.088; cross-correlation at lag − 1: − 0.666, *P* = 0.021; and cross-correlation at lag − 2: − 0.663, *P* = 0.022) (Fig. 3B). The cross-correlation analysis showed that the time series of temperature was significantly and negatively associated with the monthly prevalence of HAI, and the maximum cross-correlation coefficient was − 0.666, which was at lag − 1.

## Discussion

Since morbidity, mortality, and healthcare costs are impacted by HAI, this study aimed to analyze its prevalence at The First Affiliated Hospital, Sun Yat-sen University over a four-year period. The results indicated that the annual prevalence of HAI significantly decreased from 2.39% in 2018 to 1.41% in 2021. Interestingly, the prevalence of HAI in this study was also less than that reported in most previous studies, both domestic and foreign [2, 5, 6].

Not only the prevalence rates of HAI were greater in ICU patients who were vulnerable due to their underlying comorbidities and the presence of invasive catheters and devices, affecting nearly 30% of patients and similar to previous studies [17, 18], but HAI also occurred in nearly 30% of surgical patients in our hospital. Thus, strengthening HAI surveillance and implementing control measures in both the ICU and the surgical department are important aspects of HAI reduction.

In this study, respiratory tract infection was found to be the most common type of HAI, with an average of 44.47% over four years, a prevalence which was significantly less than that in a tertiary general hospital in Beijing (64.7%) [9]. While the reduction and control of the prevalence of respiratory tract infection should be a priority within China to reduce the prevalence of HAI, the prevalence of ventilator-associated pneumonia remained almost constant (9.70 per 1000 patient days in 2018–2021) in our study and was obviously greater than that found in hospitals in the USA (3.20 per 1000 patient days in 2015) [19].

The use of antibiotics reported in the current study revealed a prevalence of 37.6%, which was less than that in the USA (51.9%) [5] but greater than that in the European Union (30.5%) [20]. Moreover, bloodstream infection is defined by the presence of microorganisms in the blood, which might result in underreporting in many hospitals due to the high use of antibiotics that results in some blood cultures giving false-negative results. In this regard, bloodstream infection accounted for 11.59% of HAIs in this study, which was only less than the prevalence of respiratory tract infection. A systematic review of the prevalence of HAI in Mainland China has revealed, however, that the average prevalence of bloodstream infection in general hospitals in China from 2006 to 2016 was 2.65% [2], which is much less than that found in the current study. This finding might be due to the encouragement of blood culture to increase the detection rate of microbiological testing.

Furthermore, this study documented a much higher proportion of nondevice-associated urinary tract infections (69%) than catheter-associated urinary tract infections (0.53%), thus revealing the importance of infection control for nondevice-associated infections. In contrast, the most common type of HAI in a study in Germany was urinary tract infection (21.6%), which was associated with catheter use in more than 60% of cases [21]. In Germany, approximately 15–25% of all inpatients receive catheterization at least once during their hospital stay, but catheter-associated bacteriuria is usually asymptomatic, and less than 5% of cases result in bacteremia requiring treatment [21].

Given the potential impact of the COVID-19 pandemic on HAI prevention and surveillance, this study analyzed and identified potential changes in the prevalence and distribution of HAI between 2018–2019 and 2020–2021. Inconsistent with previous studies in US hospitals [22], a widespread decrease in HAI prevalence, especially that related to respiratory tract infection, has been observed in this study’s hospital since the outbreak of COVID-19 at the end of 2019.

Since the outbreak of COVID-19, most Chinese people have developed the habit of wearing face masks in public areas to prevent the spread of respiratory pathogens. Leung et al*.* have reported that surgical face mask use can significantly reduce the transmission of human coronaviruses, influenza viruses, and rhinoviruses in respiratory droplets or aerosols from symptomatic individuals [23, 24]. During the COVID-19 pandemic, the increased focus on hand hygiene, the use of personal protective equipment, environmental cleaning, and patient isolation as well as the addition of bioaerosol treatment and COVID-19 mitigation measures significantly reduced airborne ultrafine particles and altered the bioburden of hospital environments, which may have resulted in the reduction of HAI prevalence in medical institutions [13, 22]. Specifically, the HAI prevalence steadily decreased from 2018 to 2021, ranging from 2.39% in 2018 to 1.41% in 2021. To prevent the spread of disease, our hospital implemented a series of strict management measures during the COVID-19 pandemic. First, all people entering hospital areas must wear masks and are not allowed to take them off. Second, family members of patients are forbidden to visit patients in the inpatient ward, and strict management measures have been implemented for patients’ caretakers; for example, non-necessary caretakers are not allowed to stay in the ward, only one fixed caretaker is allowed to stay in the ward if necessary, and the caretakers are not allowed to walk around the ward at will. Third, additional sickbeds in inpatient wards are prohibited, and the distance between sickbeds must be strictly maintained, including 0.8 m for general wards and 1.0 m for ICU wards. Fourth, the environmental surface of the general wards is disinfected with 500 mg/L chlorine-containing disinfectant at least twice a day. Also, surface disinfection of inpatient elevators occurs once every 2 h, and air disinfection of outpatient elevators occurs twice a day. Finally, staff with a fever, respiratory tract infection, or other symptoms are not allowed to come to the hospital to work, until these symptoms disappear.

Interestingly, not only have we found that the prevalence of HAI peaked in the winter, from December to February of the following year, but our meteorological data also showed that these three months were the months with the lowest outdoor temperature in Guangzhou, China. In our study, the HAI prevalence was negatively correlated with the outdoor temperature. Similar to previous investigations, seasonal variation would affect the prevalence of respiratory tract infection on account of the cold weather, which is associated with the increased occurrence of respiratory tract infection [25, 26].

Although we found that the outdoor temperature was an important factor of the regional and seasonal factors that led to the difference in the prevalence of HAI, it was not the only factor. For example, whether the economic difference is also one of the reasons for the regional differences in HAI was not addressed. Nevertheless, there are huge discrepancies in socioeconomic conditions and the gross domestic product between different provinces and regions in China [8]; therefore, it is difficult to interpret whether the different HAI rates across regions may be related to social or economic determinants.

Consistent with respiratory tract infection, the prevalence of gastrointestinal infection, intra-abdominal infection, surgical site infection, deep surgical site infection, and intracranial infection was greater in the winter. Nevertheless, the prevalence of urinary tract infection was lower during the winter months. Gastrointestinal infection has been associated with seasonal variation because the viruses are introduced into the hospital by infected patients on admission during community outbreaks during the winter [27]. Meanwhile, previous studies have revealed that urinary tract infections have a strong pattern of seasonality, with peaks in the summer and troughs in the winter [28]. The morbidity of urinary tract infections may increase with rising temperatures. Also, dehydration and the corresponding lower urine output caused by warmer weather may be the reason for the seasonality of urinary tract infections [29].

Several limitations in this study should be mentioned. First, this study was performed in a single-center; therefore, our findings cannot be generalized to all hospitals in different regions of China. Second, the details of infection were more clearly recorded in patients with longer hospital stays compared with those with a shorter hospital stay, leading to data regarding temporary infections to be neglected. Lastly, due to the lack of relevant socioeconomic data, the influence of socioeconomic factors on HAI was not taken into account in our study.

## Conclusion

In summary, this study revealed a significantly decreased HAI prevalence since the outbreak of COVID-19, particularly in respiratory tract infections, possibly because of strengthened surveillance and implementation of control measures at the participating hospital. Additionally, the overall prevalence of HAI in our study increased significantly during the winter months. Ultimately, these results highlight the urgency of preventing HAI.

## Supplementary Information

**Additional file 1: Table S1. **Demographic and clinical characteristics of all discharged patients between 2018-2019 and 2020-2021.

## Data Availability

All data generated or analyzed during this study are included in this published article.

## Abbreviations

CI: Confidence interval
HAI: Healthcare-associated infection
ICU: Intensive care unit
IRR: Incidence rate ratio
SMD: Standardized mean difference
