# Supplementary material for: Trending prevalence of healthcare-associated infections in a tertiary hospital in China during the COVID-19 pandemic
Source: BMC Infect Dis. 2023 Jan 20;23:41. doi: 10.1186/s12879-022-07952-9 (PMC9857900; doi:10.1186/s12879-022-07952-9)
Supplement: Supplementary file 1 — Additional file 1: Table S1. Demographic and clinical characteristics of all discharged patients between 2018-2019 and 2020-2021. [file 12879_2022_7952_MOESM1_ESM.docx]

**Table S1. Demographic and clinical characteristics of all discharged patients between 2018-2019 and 2020-2021**

| **Variables** | **2018-2019** | **2020-2021** | ***P*-value** | **SMD** |
| --- | --- | --- | --- | --- |
| **Age, median (IQR)** | 47.0 (31.0,61.0) | 49.0 (33.0,62.0) | <0.001 | 0.057 |
| **Gender** |  |  |  |  |
| Female | 130519 (52.9%) | 121960 (51.6%) | <0.001 | 0.026 |
| Male | 116276 (47.1%) | 114503 (48.4%) |  |  |
| **Length of stay, median (IQR)** | 5.0 (2.0,10.0) | 5.0 (2.0,9.0) | <0.001 | 0.032 |
| **Department** |  |  | <0.001 | 0.098 |
| Internal medicine | 60521 (24.5) | 62398 (26.4) |  |  |
| Surgery | 93583 (37.9) | 87686 (37.1) |  |  |
| Gynaecology and Obstetrics | 36959 (15.0) | 29362 (12.4) |  |  |
| Pediatrics | 10549 (4.3) | 10088 (4.3) |  |  |
| Otolaryngology | 10222 (4.1) | 8956 (3.8) |  |  |
| Imaging medicine | 12863 (5.2) | 15167 (6.4) |  |  |
| Others | 22098 (9.0) | 22806 (9.6) |  |  |

Note: IQR = interquartile range. Continuous variables were compared using the rank-sum test. Categorical variables were compared by using the Chi-square test. SMD: Standardized mean difference. SMD < 0.1 was considered balance.
